# Common variants in the *ATM*, *BRCA1*, *BRCA2*, *CHEK2 *and *TP53 *cancer susceptibility genes are unlikely to increase breast cancer risk

**DOI:** 10.1186/bcr1669

**Published:** 2007-04-11

**Authors:** Caroline Baynes, Catherine S Healey, Karen A Pooley, Serena Scollen, Robert N Luben, Deborah J Thompson, Paul DP Pharoah, Douglas F Easton, Bruce AJ Ponder, Alison M Dunning, the SEARCH breast cancer study

**Affiliations:** 1 Cancer Research-UK Dept of Oncology, Cancer Research-UK Genetic Epidemiology Unit and EPIC, Strangeways Research Laboratory, Worts Causeway, Cambridge CB1 8RN, UK

## Abstract

**Introduction:**

Certain rare, familial mutations in the *ATM*, *BRCA1*, *BRCA2*, *CHEK2 *or *TP53 *genes increase susceptibility to breast cancer but it has not, until now, been clear whether common polymorphic variants in the same genes also increase risk.

**Methods:**

We have attempted a comprehensive, single nucleotide polymorphism (SNP)- and haplotype-tagging association study on each of these five genes in up to 4,474 breast cancer cases from the British, East Anglian SEARCH study and 4,560 controls from the EPIC-Norfolk study, using a two-stage study design. Nine tag SNPs were genotyped in *ATM*, together with five in *BRCA1*, sixteen in *BRCA2*, ten in *CHEK2 *and five in *TP53*, with the aim of tagging all other known, common variants. SNPs generating the common amino acid substitutions were specifically forced into the tagging set for each gene.

**Results:**

No significant breast cancer associations were detected with any individual or combination of tag SNPs.

**Conclusion:**

It is unlikely that there are any other common variants in these genes conferring measurably increased risks of breast cancer in our study population.

## Introduction

Four of the genes which lie in the DNA damage-recognition and repair pathway, *ATM*, *BRCA1*, *BRCA2 *and *TP53*, have mutations that are recognised to increase breast cancer susceptibility with moderate to high penetrance. Such mutations are very rare, and most probably of recent origin. A fifth gene, *CHEK2*, in the same pathway, has a deletion (1100delC) that reaches polymorphic frequencies (>0.01) in some European countries and doubles the risk of breast cancer in female carriers [1]. Together these mutations account for only a small proportion (2% to 5%) of all breast cancer incidences [2,3]. Breast cancer is, however, a common disease and genetic epidemiological data suggest that there is a low-penetrance genetic contribution to most cases [4,5]. It is likely that at least a part of breast cancer aetiology will fit the common disease-common variant hypothesis, which states that patients with a common, complex disease are likely to share some common, low-penetrance alleles that increase their susceptibility to that disease. This raises the question of whether such common, polymorphic susceptibility alleles exist within these five genes in addition to the rare, disease-causing mutations that are already known.

It is now possible to attempt a comprehensive exclusion of all common single nucleotide polymorphisms (SNPs) from association with breast cancer susceptibility, using an empirical tag SNP approach. Studies have already been attempted in some of these genes. In *ATM*, Tamimi and colleagues [6] examined 5 haplotype-tagging SNPs (htSNPs) in approximately 3,000 subjects from the Nurses Heath Study but found no associations with breast cancer risk. For *BRCA1*, Cox and colleagues [7] used 4 htSNPs in the Nurses Health Study and identified one haplotype associated with an odds ratio (OR) of 1.18 (95% confidence interval (CI) 1.02 to 1.37), while Freedman and colleagues [8] examined 9 SNP-tagging SNPs (stSNPs) in the approximately 900 Caucasian subjects from the Multi-Ethnic Cohort but found no significant effects. For *BRCA2*, Freedman and colleagues [9] also used 21 stSNPs in the Multi-Ethnic Cohort and found one (rs206340) to be associated with a homozygous increase in risk (OR = 1.59, 95% CI 1.18 to 2.16). In addition, numerous studies have examined the N372H amino acid substitution in *BRCA2 *with conflicting results [10-12]. In *CHEK2*, Einarsdottir and colleagues [13] studied 6 htSNPs in approximately 3,000 Swedish cases and controls but found no significant associations with breast cancer. No comprehensive tagging study has yet been carried out for *TP53*, but several studies have examined the association of the non-synonymous R72P change and again the results have been mixed (reviewed in [14]).

We have used a combined SNP- and haplotype-tagging approach in an attempt to mark all the common variants in these five genes. Selected tag SNPs were then evaluated in our East Anglian breast cancer case-control study. The principal hypothesis underlying this experiment is that one or more common variants in these five genes are associated with an altered risk of breast cancer. We therefore aimed to identify a set of tag SNPs that efficiently captures all the known common variation (minor allele frequency (MAF) > 0.05) and is, therefore, likely also to tag most of the unknown common variants. This approach is most reliable where the gene has been re-sequenced in a sample of individuals that is sufficiently large to identify all common variants. We thus preferentially used re-sequencing data from the Environmental Genome Project (EGP) [15], but in genes where data was not available at the commencement of our study, data from the HAPMAP project [16] was used. HAPMAP does not re-sequence genes but provides genotype data on a sufficiently dense set of SNPs in samples of subjects from different ethnicities.

Our goal was to identify any common variants that show evidence for association with breast cancer susceptibility or, failing that, to exclude the possibility that common variants in these five genes are associated with an altered risk of breast cancer.

## Materials and methods

### Patients and controls

Cases were drawn from the SEARCH (breast) collection, an ongoing population-based study of breast cancer, with cases ascertained through the Eastern Cancer Registry (formerly East Anglian Cancer Registry). All patients diagnosed with invasive breast cancer below age 55 years since 1991 and still alive in 1996 (prevalent cases, median age 48 years), together with all those diagnosed below age 70 years between 1996 and the present (incident cases, median age 54 years), were eligible to take part. Of the eligible breast cancer patients, 67% returned a questionnaire and 64% provided a blood sample for DNA analysis. Eligible patients who did not take part in the study were similar to participants except, as might be expected, the proportion of clinical stage III/IV cases was somewhat higher in non-participants (Additional file 1). Controls were randomly selected from the Norfolk component of EPIC (European Prospective Investigation of Cancer). EPIC is a prospective study of diet and cancer being carried out in nine European countries. The EPIC-Norfolk cohort comprises 25,000 individuals resident in Norfolk, East Anglia, the same region from which the cases have been recruited. Controls are not matched to cases, but are broadly similar in age, being aged 42 to 81 years. The ethnic background of both cases and controls, as reported on the questionnaires, is similar, with >98% being white. This study has been approved by the Eastern Region Multicentre Research Ethics Committee, and all participants gave written informed consent.

The total number of cases available for analysis was 4,474, of which 27% were prevalent cases. The samples have been split into two sets in order to save DNA and reduce genotyping costs: the first set (*n *= 2,271 cases and 2,280 controls) is genotyped for all SNPs and the second set (*n *= 2,203 cases and 2,280 controls) is then tested for those SNPs that show marginally significant associations in set 1 (P-heterogeneity or P-trend <0.1). Cases were randomly selected for set 1 from the first 3,500 recruited, with set 2 comprising the remainder of these plus the next 974 incident cases recruited. As the prevalent cases were recruited first, the proportion of prevalent cases was somewhat higher in set 1 than in set 2 (33% versus 20%). Median age at diagnosis was similar in both sets (51 and 52 years old, respectively). There was no significant difference in the morphology, histopathological grade or clinical stage of the cases by set or by prevalent/incident status.

### Power

The statistical power of the study depends on the susceptibility allele frequency, the risks conferred and the genetic mode of action (dominant, recessive, co-dominant). The staged approach substantially reduces genotyping costs without significantly affecting statistical power – a comparison is shown in Additional file 2. For example, assuming that the causative SNP is tagged with a pairwise correlation coefficient (r_p_^2^) of 0.8, a type I error rate of 0.0001 and a genotyping success rate of 0.95, the staged/full study has 86/88% power to detect a dominant allele with MAF = 0.05 that confers a relative risk of 1.5 or 87/89% power to detect a dominant allele with MAF = 0.25 that confers a relative risk of 1.3. Power to detect recessive alleles is less; 53/60% for an allele with MAF = 0.25 and risk 1.5 and 71/75% for an allele with MAF = 0.5 and risk 1.3.

### Selection of tagging SNPs

We attempted to define a set of tag SNPs such that all known common SNPs in a gene had an estimated r^2 ^> 0.8 with at least one tag SNP using the *tagSNPs *program [17]. The best measure of the extent to which one SNP tags another SNP is the pairwise correlation coefficient (r_p_^2^), since the loss in power incurred by using a marker SNP in place of a true causal SNP is directly related to this measure. We aimed to define a set of tagging SNPs such that all known common SNPs had an estimated r_p_^2 ^of >0.8 with at least one tagging SNP. However, some SNPs are poorly correlated with other single SNPs but may be efficiently tagged by a haplotype defined by multiple SNPs, thus reducing the total number of tag SNPs needed. As an alternative, therefore, we aimed for a correlation between each SNP and a haplotype of tag SNPs (r^2^S) of >0.8. Since tag SNP selection is problematic if there is extensive haplotype diversity, where necessary we divided a gene into haplotype blocks and selected the tagging SNPs for each block separately. It is possible to use a variety of formal definitions of haplotype blocks, but we simply used the graphical representations of the pattern of linkage disequilibrium (LD) based on D' and selected blocks such that the common haplotypes in each block accounted for at least 80% of all haplotypes observed using the Haploview program.

This tag SNP approach is most reliable where the gene has been re-sequenced in a sample of individuals that is sufficiently large to identify all common variants. We preferentially used data from the EGP [15], which has been re-sequencing candidate genes for cancer across panels of individuals representative of US ethnicities. The original panel (P1-PDR90) of 90 individuals consisted of 24 European Americans, 24 African Americans, 12 Mexican Americans, 6 Native Americans and 24 Asian Americans, but the ethnic group identifiers were not available. It is known that there is greater genetic and haplotype diversity in individuals of African origin. To reduce this we have identified and excluded 28 of the samples with the greatest African ancestry by comparing the genotypes of the PDR-90 subjects with the genotypes of the National Heart Lung and Blood Institute Variation Discovery Resource Project African American Panel [15] for the same SNPs. Data from the remaining 62 individuals were used to identify tag SNPs. For *CHEK2*, where complete EGP data were not initially available, data from European (CEU) subjects in HAPMAP were used as an alternative [16].

The *CHEK2 *Del1100C mutation was too rare to be selected as a tag SNP, or to be tagged in the current study, but it had been previously assayed in the SEARCH cases and controls [1] and those data have been incorporated here.

### Taqman genotyping

Genotyping was carried out using Taqman^® ^(Applied Biosystems, Warrington, UK) according to the manufacturer's instructions. Primers and probes were supplied directly by Applied Biosystems as Assays-by-Design™. All assays were carried out in 384-well plates. Cases and controls were arranged in a chequerboard pattern on each plate to ensure even treatment during the assay procedure and each plate included negative controls (with no DNA) and positive controls duplicated on a separate quality control plate. Plates were read on the ABI Prism 7900 using the Sequence Detection Software (Applied Biosystems). Failed genotypes were not repeated. Assays in which the genotypes of duplicate samples did not show >95% concordance were discarded and replaced with alternative assays with the same tagging properties.

### Statistical methods

For each SNP, deviation of genotype frequencies in controls from the Hardy-Weinberg equilibrium (HWE) was assessed by a χ^2 ^test with one degree of freedom. Genotype frequencies in cases and controls were compared by χ^2 ^test for heterogeneity (two degrees of freedom) and test for trend (one degree of freedom). Genotype specific risks were estimated as ORs using unconditional logistic regression. Genotype distributions were also compared between prevalent and incident cases and between subjects in set 1 and set 2 with χ^2 ^tests (two degrees of freedom). No statistically significant differences were found (data not shown) and so the results have been combined. The *tagSNPsv2 *program [17] was used to impute all the haplotypes generated by the tag SNPs in each LD block and to estimate the probabilities of each subject carrying each of the common (>0.05) haplotypes. Rarer haplotype probabilities were pooled into a single category. Haplotype frequencies were compared between cases and controls and haplotype-specific risks were estimated as ORs with associated CIs using unconditional logistic regression.

## Results and discussion

### SNP-tagging

#### ATM

The EGP identified 75 common variants in their set of 90 mixed-ethnicity subjects. After the exclusion of the 28 subjects with most African ancestry, 69 SNPs suitable for study in our European population remained. These fall into a single LD block with no evidence for recombination hot-spots. A set of nine tag SNPs were identified using the *tagSNPsv2 *program [17] and all were successfully genotyped. Tagging details are shown in Table 1.

#### BRCA1

The EGP identified 123 common variants, of which 113 were suitable for study in our sets (Table 1). Again, these lie in a single block of LD. A set of eight tag SNPs was identified but three of these could not be made into Taqman^® ^assays and no others could be found to provide the same information (that is, they are singletons). A further SNP appeared to be hyper-mutable and it was not further investigated. Eventually, four tag SNPs were successfully genotyped (Table 1). An additional SNP, Q356R (*BRCA1-02*), was not selected as a tag SNP as its MAF in EGP was below our threshold of 0.05, but it was genotyped here because we had previously found some evidence for its association with an increased risk of both ovarian and breast cancer.

#### BRCA2

**Figure 1:**
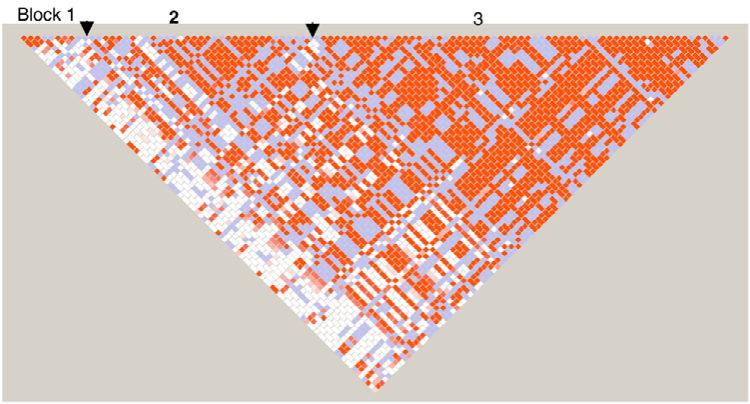
Haploview output showing linkage disequilibrium relationships between the 91 eligible *BRCA2 *SNPs. The matrix indicates the D' value between each pair of SNPs – darker colours indicate higher values.

The EGP identified 113 common SNPs and 91 of these remained for study after exclusion of the African subjects. The *BRCA2 *block structure (Figure 1) is more complex than seen in the other genes; the first ten SNPs show no clear block structure and the remaining can be divided into two, largely separate, blocks. Twenty-two tag SNPs were initially identified but for six of these (all singletons) the local sequence precluded the manufacture of a working Taqman^® ^assay and so these could not be analysed here (Table 1).

#### CHEK2

At the commencement of this study this gene had not been re-sequenced by EGP and so HAPMAP phase 1 data for the Caucasians (CEPH trios) were used instead. HAPMAP had genotypes for 30 SNPs with MAF > 0.05 and these fell into a single LD block. Ten tag SNPs (Table 1), were successfully genotyped. Previously published [1] data on a subset of these subjects who were genotyped for the 1100delC variant are also included for comparison (Table 2).

#### TP53

The EGP identified 46 common SNPs, of which 39 were suitable for study after exclusion of the African subjects. These lie in a single LD block and are tagged with six SNPs. We set the tagging parameters to include two SNPs that had been selected for a previous study (TP53-01 (R72P, rs1042522) and TP53-02 (rs1625895)). One SNP (rs17880722) could not be made into a Taqman^® ^assay and, since these were singletons, this left a final set of five tag SNPs that were genotyped (Table 1).

### SNP associations

The genotype distributions for all SNPs genotyped in breast cancer case-control set 1 are shown in the left-hand columns of Table 2. In the *BRCA1 *and *TP53 *genes, no SNPs showed suggestive association (*p *< 0.1) at this stage and none were further investigated. One tag SNP in the *ATM *gene (ATM-08, rs3092991), four in *BRCA2 *(BRCA2-04,-11,-13 and -21/rs206118, rs11571686, rs1799955 and rs206343) and three in *CHEK2 *(CHEK2-09,-11 and -14/rs2236141, rs1076807 and rs9608698) provided sufficient evidence of association to merit further evaluation in case-control set 2. The genotype distributions of these SNPs in the entire study (sets 1 and 2) are shown in the right-hand columns of Table 2. After the completion of both stages, no tag SNP from *ATM*, *BRCA1*, *BRCA2 *or *TP53 *was associated with a significant difference in breast cancer risk.

The only SNP that was significantly associated (nominal threshold of *p *< 0.05) after completion of stage 2 was *CHEK2-14 *(rs9608698; P-trend = 0.02). This SNP was associated with an increased risk of breast cancer in both heterozgotes (OR = 1.10; 95% CI 0.99 to 1.21) and rare homozygotes (OR = 1.15; 95% CI 1.02 to 1.29) relative to the common homozygotes.

We specifically chose to study SNPs that generate amino acid substitutions and had been included in previously published association studies. *BRCA1 *P271L (*BRCA1-01*, rs1799917), *BRCA2 *N372H (*BRCA2-22*, rs144848) and *TP53 *R72P (TP53-01, rs1042522) were included in the set of tagging SNPs for their respective genes and it can be seen that, in this study, none of these SNPs show any significant association with breast cancer risk. A recent collaborative study [18] has also investigated *BRCA2 *N372H in more than 31,000 subjects (cases and controls) as well as *TP53 *R72P in almost 20,000 subjects. That study, similarly, found no main effect with either amino acid substitution, although it did report some evidence of *BRCA2 *HH homozygotes in the youngest age groups having an increased risk of breast cancer. This finding is compatible with the original two publications, which reported a significantly increased risk of breast cancer in HH homozygotes, since both had concentrated on very young onset breast cancer cases [11,12].

We were unable to confirm the previously reported breast cancer association of *BRCA1 *Q356R (*BRCA1-02*, rs1799950). The OR for the rare, RR, homozygote relative to the common, QQ, group is 0.63 (95% CI 0.23 to 1.23; P-trend = 0.20, P-recessive test = 0.18). This estimate is comparable with our previous estimate, which was based on only 1,400 subjects [19], but even this study, which is larger by 6 times, has <50% power to test the hypothesis that this SNP is associated with such a recessive protective effect (Additional file 2).

### Haplotype associations

Some of the variants within these genes have not been well tagged by an individual tag SNP, but a number of these have been better covered by a combination (haplotype) of several SNPs. Thus, the haplotypes predicted from the genotypes of tag SNPs were also tested for association with breast cancer risk; each common haplotype (predicted frequency >0.05) in each LD block was tested individually, in addition to the effect of the combined rarer haplotypes. The results are shown in Table 3.

For *TP53 *four common haplotypes are predicted to be formed from the five tag SNPS and none are significantly associated with differences in breast cancer risk. For *BRCA1 *the five genotyped SNPs generate five common haplotypes. Four of these are not associated with differences in breast cancer risk. The remaining one uniquely carries the R356 allele of the Q356R polymorphism and its OR is thus very similar to that calculated on the Q356R SNP analysis. For *ATM*, the eight tag SNPs generate five common haplotypes. Four show no significant association with breast cancer risk and the remaining one, which uniquely carries the rare allele of *ATM*-08 (rs3092991), displays a similar OR to that calculated by examination of this SNP alone. Both the potential associations with *BRCA1 *Q356R and *ATM *rs3092992 were found to be false positives after examination in stage 2 and the same conclusion can be drawn about their haplotypes.

For *BRCA2 *the pattern is more complex and haplotypes were analysed separately in the three LD blocks. In each block certain haplotypes gave results in set 1 that merited further investigation in set 2 (Table 3). Three further tag SNPs, in addition to the five described in the previous section, were genotyped in set 2 to enable the discrimination of these haplotypes. However, after stage 2, the potential associations with these haplotypes had reduced in significance and so we can conclude that they were also false positives.

Freedman and colleagues [9] reported that a haplotype of *BRCA2 *tagged by SNP rs206340 was associated with a significant increase in breast cancer risk among homozygotes (OR aa/gg = 1.59; 95% CI 1.18 to 2.16)). In our study the equivalent tag SNP was *BRCA2-21 *(rs206343; r_p_^2 ^with rs206340 = 0.89), which contrarily showed a non-significant trend for the minor allele to decrease, rather than increase, risk (Table 2; OR gg/aa = 0.89; 95% CI 0.73 to 1.09)].

Two haplotypes of *CHEK2*, both carrying the minor allele of *CHEK2-14 *(rs9608698), showed potential associations but with risks in opposing directions. These were evaluated further by generating haplotypes from the SNP markers that had already been taken into stage 2 as well as the 1100delC mutation that had been typed for a previous study. The 1100delC mutation, which has a frequency of 0.4% in these East Anglian controls, is carried on a single haplotype that also carries *CHEK2-14*. This rare haplotype is, as expected, significantly associated with breast cancer susceptibility (OR = 2.66; 95% CI 1.40 to 5.05) for the carrier versus the non-carrier, but other, more common haplotypes tagged by *CHEK2-*14 but not 1100delC also show marginal evidence for association with differences in risk

### Deviation from Hardy-Weinberg equilibrium

The coding region of the *ATM *gene has been reported to be less polymorphic than other comparable genes, indicative of constraint by Darwinian selection pressure [20]. The genotype distribution of the *BRCA2 *N372H SNP has also previously been noted to deviate from HWE [11,21], again indicating possible selection. Here we note that there are more deviations from HWE than would be expected by chance. The genotype distributions of 11 of the 45 tag SNPs (24%) deviate from HWE below the 10% significance level, and 5 (11%) deviate below the 5% level, and 1 of these (*BRCA2-18*, rs11571789) deviates with *p *= 6 × 10^-5^. All significant deviations from HWE fall in just three of the five genes, *ATM*, *BRCA2 *and *TP53*. Examination of the genotype clusters and other quality control measures gives no indication that these deviations are the result of genotyping artefacts. This leaves open the possibility that common variants in these three genes are subject to selection, acting either directly or indirectly via selective sweep across other variants in LD with them.

### Limitations

There are three classes of variant that we cannot be certain we have excluded from association. The first class comprises dominant alleles with MAF below 0.01 – these include the known rare, disease-causing mutations in these five genes. The BIC database [22] lists 27 mis-sense mutations in *BRCA1 *and 20 in *BRCA2 *(as well as many more variants of unknown function), which, like SNPs, all result from single-base substitutions, but are present only in single families. Similarly, there are as yet unknown numbers of rare mutations in the *ATM *gene that increase risk of breast cancer in heterozygous carriers [23]. Some of these result from single base substitutions and could be expected to be present in up to 3% of our cases (for example, S49C) [24].

The second class comprises those SNPs that have MAF = 0.05 to 0.25 but are recessive in effect. For both the above classes, we had insufficient statistical power, even with a sample size of more than 9,000 subjects, for exclusion of all variants by genetic association.

The third class comprises variants that are not well tagged by any of our genotyped tag SNPs or their combined haplotypes. As can be seen from Table 1, there are several of these, predominantly in the *BRCA2 *and *CHEK2 *genes. Such hard-to-tag variants often lie outside LD blocks or are hyper-mutable. The minor alleles of these SNPs do not share identity-by-descent with other SNPs and so cannot be detected by association; they will only have an effect on breast cancer risk if they are the functional and directly increase cancer susceptibility.

## Conclusion

There is ample evidence that rare mutations in each of the five genes *ATM*, *BRCA1*, *BRCA2*, *CHEK2 *and *TP53 *cause increased susceptibility to breast cancer in the families who carry them, but we find no evidence for the existence of common, polymorphic susceptibility alleles in these genes. However, there remains good evidence that such alleles, conforming to the common disease-common variant hypothesis, do exist in other breast cancer susceptibility genes.

## Abbreviations

CI = confidence interval; EGP = Environmental Genome Project; ht-SNP = haplotype tagging SNP; HWE = Hardy-Weinberg equilibrium; LD = linkage disequilibrium; MAF = minor allele frequency; OR = odds ratio; SNP = single nucleotide polymorphism; st-SNP = SNP tagging SNP.

## Competing interests

The authors declare that they have no competing interests.

## Authors' contributions

CB, CSH, KAP and SS chose the tagging set of SNPs, carried out the assays, analysed the data and helped prepare the manuscript. RNL provided data on the EPIC subjects. DJT chose and developed a suite of tag SNP and haplotype deduction programs for SEARCH and supervised their appropriate use. AMD, PDPP, DFE and BAJP are principle investigators within SEARCH. AMD managed this project and prepared the manuscript. CB, CSH and KAP contributed equally to this study. The SEARCH study team are currently: Jean Abraham, Shahana Ahmed, Antonis Antoniou, Patrick Benusiglio, Fiona Blows, Arancha Cebrian, Don Conroy, Bridget Curzon, Gary Dew, Kristy Driver, Helen Field, Patricia Harrington, Clare Jordan, Fabienne Lesueur, Craig Luccarini, Rebecca Mayes, Hannah Munday, Barbara Perkins, Karen Redman, Mitul Shah, Jonathan Tyrer, Paula Smith and Judy West.

## Supplementary Material

Additional file 1 A table listing the basic epidemiological details of the breast cancer cases and controls in set 1 and set 2. Click here for file

Additional file 2 A table showing a statistical power comparison of the two-stage versus the single-stage study design. Click here for file

## Figures and Tables

**Table 1:** SNP tagging details and data for each gene

Gene	SNP source	Total SNPs with MAF > 0.05	Total SNPs after exclusion of African subjects	No. of Tag SNPs selected for r^2 ^> 0.8	Usable SNPs	No. of SNPs tagged with r_p_2 > 0.8 (percent)	No. of SNPs tagged with r_p_^2 ^> 0.5 (percent)	No. of SNPs tagged with r^2^S > 0.8 (percent)
*ATM*	EGP	75	69	9	9	64 (92)	69 (100)	65 (94)
*BRCA1*	EGP	123	113	8	5	67 (59)	109 (96)	77 (68)
*BRCA2*	EGP	113	91	22	16	53 (58)	86 (95)	61 (67)
*CHEK2*	HAPMAP	30	N/A	10	10	16 (53)	20 (67)	22 (73)
*TP53*	EGP	46	39	6	5	24 (61)	35 (90)	31 (79)

EGP, NIEHS Environmental Genome Project; MAF, minor allele frequency; SNP, single nucleotide polymorphism.

**Table 2:** Genotype distributions of all tag SNPS in case-control set 1 and the eligible ones in sets 1 and 2 combined

			Set 1						Sets 1 and 2			
				
SNP	MAF	Base change	Controls	Cases	Odds ratio (95 percent CI)	HWE *p *value	Genotype frequency *p *value	Trend test *p *value	Controls	Cases	Odds ratio (95 percent CI)	Trend test *p *value
***ATM***												
		GG	1,881	1,808	1^a^							
ATM-04	0.09	GT	378	364	1.00 [0.86–1.17]	0.84	0.84	0.79				
rs4987876		TT	18	21	1.21 [0.64–2.29]							
		AA	707	694	1^a^							
ATM-05	0.44	AG	1,146	1,069	0.95 [0.83–1.09]	0.24	0.52	0.84				
rs189037		GG	420	427	1.04 [0.87–1.23]							
		AA	2,103	1,992	1^a^							
ATM-06	0.04	AG	169	183	1.14 [0.92–1.42]	0.19	0.47	0.29				
rs2234996		GG	6	5	0.88 [0.27–2.89]							
		AA	727	713	1^a^							
ATM-07	0.43	AG	1,147	1,058	0.94 [0.82–1.07]	0.14	0.37	0.80				
rs599164		GG	398	409	1.05 [0.88–1.24]							
		AA	1,627	1,611	1^a^				3,280	3,223	1^a^	
ATM-08	0.15	AG	606	553	0.92 [0.81–1.05]	0.03	0.09	0.06	1,193	1,081	0.92 [0.84–1.01]	0.10
rs3092991		GG	44	27	0.62 [0.38–1.01]				82	74	0.92 [0.67–1.26]	
		GG	1,052	990	1^a^							
ATM-09	0.32	GA	1,000	971	1.03 [0.91–1.17]	0.48	0.70	0.41				
rs665293		AA	222	227	1.09 [0.89–1.33]							
		TT	712	688	1^a^							
ATM-10	0.43	TA	1,110	1,036	0.97 [0.84–1.11]	0.45	0.34	0.45				
rs582157		AA	405	426	1.09 [0.92–1.29]							
		CC	2,043	1,990	1^a^							
ATM-11	0.05	CT	214	183	0.88 [0.71–1.08]	0.01	0.35	0.15				
rs1800889		TT	13	9	0.71 [0.30–1.67]							
		GG	1,945	1,872	1^a^							
ATM-12	0.08	GA	310	300	1.01 [0.85–1.19]	0.02	0.48	0.64				
rs611018		AA	22	14	0.66 [0.34–1.30]							
***BRCA1***												
P871l		TT	1,001	939	1^a^							
BRCA1-01	0.33	TC	1,031	996	1.03 [0.91–1.17]	0.19	0.40	0.22				
rs1799917		CC	234	252	1.15 [0.94–1.40]							
Q356R		TT	2,004	1,955	1^a^				4,013	3,879	1^a^	
BRCA1-02	0.06	TC	256	221	0.89 [0.73–1.07]	0.13	0.15	0.08	511	469	0.95 [0.83–1.08]	0.23
rs1799950		CC	13	6	0.47 [0.18–1.25]				23	14	0.63 [0.32–1.23]	
		TT	1,673	1,580	1^a^							
BRCA1-05	0.14	TC	553	568	1.09 [0.95–1.25]	0.87	0.45	0.38				
rs8176166		CC	47	42	0.95 [0.62–1.44]							
		TT	1,281	1,208	1^a^							
BRCA1-06	0.25	TG	857	851	1.05 [0.93–1.19]	0.69	0.68	0.65				
rs8176199		GG	137	127	0.95 [0.76–1.27]							
		GG	1,893	1,834	1^a^							
BRCA1-07	0.09	GA	362	337	0.96 [0.82–1.13]	0.31	0.53	0.38				
rs3737559		AA	22	15	0.70 [0.36–1.36]							
***BRCA2***												
		TT	715	688	1^a^							
BRCA2-01	0.45	TC	1,083	1,057	1.01 [0.89–1.16]	0.08	0.72	0.62				
rs206115		CC	475	435	0.95 [0.81–1.13]							
		GG	870	813	1^a^				1,676	1,621	1^a^	
BRCA2-02	0.39	GA	1020	1,010	1.06 [0.93–1.21]	0.39	0.63	0.79	2,145	2,028	0.98 [0.89–1.07]	0.95
rs206116		AA	380	355	1.00 [0.84–1.19]				724	705	1.01 [0.89–1.14]	
		GG	1,576	1,504	1^a^							
BRCA2-03	0.17	GA	628	601	1.00 [0.88–1.15]	0.96	0.64	0.58				
rs3092989		AA	63	71	1.18 [0.83–1.67]							
		AA	1,515	1,506	1^a^				3,046	2,943	1^a^	
BRCA2-04	0.19	GA	667	616	0.93 [0.81–1.06]	0.07	0.02	0.02	1,328	1,287	1.00 [0.92–1.10]	0.29
rs206118		GG	91	57	0.63 [0.45–0.88]				172	132	0.79 [0.63–1.00]	
		AA	1,353	1,241	1^a^				2,691	2,502	1^a^	
BRCA2-05	0.22	GA	763	719	1.03 [0.90–1.17]	0.09	0.75	0.98	1,594	1,486	1.00 [0.92–1.10]	0.40
rs206119		GG	102	86	0.92 [0.68–1.24]				203	216	1.14 [0.94–1.40]	
		GG	1,263	1,189	1^a^							
BRCA2-07	0.25	GT	875	828	1.01 [0.89–1.14]	0.31	0.21	0.23				
rs9567552		TT	135	158	1.24 [0.98–1.59]							
		GG	612	626	1^a^				1,228	1,200	1^a^	
BRCA2-09	0.48	GA	1,137	1,082	0.93 [0.81–1.07]	0.46	0.22	0.08	2,317	2,189	0.97 [0.88–1.07]	0.60
rs9534174		AA	523	461	0.86 [0.72–1.02]				1,006	954	0.97 [0.86–1.09]	
		CC	1,487	1,453	1^a^							
BRCA2-10	0.18	CT	669	620	0.95 [0.83–1.08]	0.62	0.63	0.73				
rs2126042		TT	70	74	1.08 [0.77–1.51]							
		AA	1,824	1,701	1^a^				3,609	3,434	1^a^	
BRCA2-11	0.10	AC	428	450	1.13 [0.97–1.31]	0.40	0.10	0.04	893	876	1.03 [0.92–1.14]	0.28
rs11571686		CC	25	35	1.50 [0.89–2.52]				52	63	1.27 [0.88–1.84]	
		AA	1,422	1,290	1^a^				2,812	2,672	1^a^	
BRCA2-13	0.21	AG	761	776	1.12 [0.99–1.27]	0.70	0.03	0.01	1,548	1,491	1.01 [0.93–1.11]	0.48
rs1799955		GG	92	115	1.38 [1.04–1.83]				194	201	1.09 [0.89–1.34]	
		GG	1,204	1,205	1^a^							
BRCA2-14	0.27	GA	907	833	0.92 [0.81–1.04]	0.83	0.26	0.10				
rs1151742		AA	167	145	0.87 [0.68–1.10]							
		AA	1,733	1,603	1^a^							
BRCA2-16	0.12	AG	484	414	0.92 [0.80–1.07]	0.33	0.56	0.43				
rs4942485		GG	34	33	1.05 [0.65–1.70]							
		CC	2,020	1,910	1^a^							
BRCA2-18	0.06	CA	235	253	1.14 [0.94–1.38]	6 × 10^-5^	0.27	0.11				
rs11571789		AA	19	24	1.34 [0.73–2.45]							
		AA	1,247	1,186	1^a^							
BRCA2-19	0.26	AG	829	820	1.04 [0.92–1.18]	0.11	0.83	0.63				
rs542551		GG	164	159	1.02 [0.81–1.29]							
		AA	1,383	1,370	1^a^				2,791	2,668	1^a^	
BRCA2-21	0.22	AG	769	720	0.94 [0.83–1.07]	0.39	0.15	0.08	1,527	1,465	1.00 [0.92–1.10]	0.54
rs206343		GG	119	90	0.76 [0.57–1.01]				225	192	0.89 [0.73–1.09]	
N372H		AA	1,149	1,109	1^a^				2,306	2,182	1^a^	
BRCA2-22	0.28	AC	942	896	0.98 [0.87–1.11]	0.07	0.96	0.78	1,892	1,824	1.02 [0.93–1.11]	0.58
rs144848		CC	171	161	0.97 [0.77–1.23]				339	333	1.04 [0.88–1.22]	
***CHEK2***												
		AA	914	857	1^a^							
CHEK2-05	0.37	AT	1,045	1,019	1.04 [0.92–1.18]	0.55	0.82	0.60				
rs5762760		TT	315	306	1.04 [0.86–1.24]							
		AA	1,296	1,243	1^a^							
CHEK2-06	0.25	AG	813	789	1.01 [0.89–1.15]	0.11	0.72	0.72				
rs2073327		GG	152	133	0.91 [0.71–1.17]							
		GG	1,554	1,548	1^a^							
CHEK2-07	0.17	GC	633	558	0.88 [0.77–1.01]	0.87	0.20	0.12				
rs2347443		CC	66	62	0.94 [0.66–1.34]							
		GG	1,150	1,130	1^a^							
CHEK2-08	0.29	GA	938	868	0.94 [0.83–1.07]	0.63	0.64	0.50				
rs738722		AA	182	175	0.98 [0.78–1.22]							
		GG	1,718	1,598	1^a^				3,439	3,199	1^a^	
CHEK2-09	0.13	GA	510	553	1.17 [1.02–1.34]	0.31	0.04	0.18	1,021	1,087	1.14 [1.04–1.26]	0.07
rs2236141		AA	41	29	0.76 [0.47–1.23]				86	69	0.86 [0.63–1.19]	
		GG	1,235	1,198	1^a^							
CHEK2-10	0.26	GA	872	834	0.99 [0.87–1.12]	0.55	0.76	0.54				
rs4035540		AA	144	127	0.91 [0.71–1.17]							
		AA	1,914	1,885	1^a^				3,866	3,770	1^a^	
CHEK2-11	0.08	AT	331	281	0.86 [0.73–1.02]	0.71	0.22	0.10	633	563	0.91 [0.81–1.03]	0.11
rs1076807		TT	17	15	0.90 [0.45–1.80]				28	23	0.84 [0.48–1.46]	
		GG	1,150	1,130	1^a^							
CHEK2-12	0.29	GA	928	851	0.93 [0.82–1.06]	0.87	0.54	0.47				
rs1807609		AA	184	178	0.98 [0.79–1.23]							
		GG	1,992	1,886	1^a^							
CHEK2-13	0.06	GA	270	2,866	1.12 [0.94–1.34]	0.19	0.38	0.17				
rs17508019		AA	5	7	1.48 [0.47–4.67]							
		CC	694	626	1^a^				1,410	1,249	1^a^	
CHEK2-14	0.45	CG	1,114	1,057	1.05 [0.92–1.21]	0.09	0.18	0.07	2,188	2,123	1.10 [0.99–1.21]	0.02
rs9608698		GG	463	488	1.17 [0.99–1.38]				940	956	1.15 [1.02–1.29]	
1100delC^b^	0.02	CC					0.90		2,887	2,671	1^a^	0.002
		delC							14	34	2.63 [1.41–4.90]	
***TP53***												
R72P		GG	1,177	1,107	1^a^							
TP53-01	0.27	GC	854	768	0.96 [0.84–1.09]	0.52	0.75	0.48				
rs1042522		CC	166	148	0.95 [0.75–1.20]							
		CC	1,622	1,545	1^a^							
TP53-02	0.14	CT	520	449	0.91 [0.79–1.05]	0.09	0.39	0.20				
rs1625895		TT	55	48	0.92 [0.62–1.36]							
		CC	1,797	1,699	1^a^							
TP53-03	0.11	CG	438	453	1.09 [0.94–1.27]	0.04	0.45	0.41				
rs2287499		GG	39	34	0.92 [0.58–1.47]							
		AA	2,053	1,971	1^a^							
TP53-06	0.05	AG	217	204	0.98 [0.80–1.20]	0.77	0.91	0.94				
rs17887200		GG	5	6	1.25 [0.38–4.10]							
		CC	2,067	1,986	1^a^							
TP53-07	0.05	CT	204	198	1.01 [0.82–1.24]	0.99	0.81	0.76				
rs17886760		TT	5	7	1.46 [0.46–4.60]							
		CC	865	833	1^a^							
TP53-08	0.39	CT	1,069	1,022	0.99 [0.87–1.13]	0.69	0.95	0.89				
rs9893249		TT	342	336	1.02 [0.84–1.22]							

Values in the minor allele frequency (MAF) and Hardy-Weinberg equilibrium (HWE) *p *value columns refer to controls only. ^a^Reference group. ^b^Attempted in only 61% of subjects. CI, confidence interval.

**Table 3:** Predicted haplotypes from the tagging SNPs for each gene and their association with breast cancer

	Set 1					Sets 1 and 2				
		
Gene	Haplotype	Frequency	*P *value	OR	95 percent CI	Haplotype	Frequency	*P *value	OR	95 percent CI
***ATM***										
04,05,06,07,08,09,10,11,12	h010100100	0.30	0.2							
	h000001000	0.28	0.7							
	h000010000	0.14	0.05	0.9	0.78–0.99					
	h100000000	0.09	0.6							
	h010100101	0.07	0.9							
	h010100110	0.05	0.2							
	Combined rare		0.4							
***BRCA1***										
02,01,05,07,06	h00000	0.60	0.7							
	h01101	0.14	0.3							
	h01000	0.09	0.2							
	h01011	0.09	0.2							
	h10000	0.06	0.08	0.9	0.71–1.02					
	Combined rare		0.5							
***BRCA2***										
Block 1: 01,02,03,04,05	h10000	0.42	0.6			h_0_00	0.42	0.5		
	h01000	0.17	0.007	1.2	0.78–0.98	h_1_00	0.17	0.2		
	h01010	0.17	0.02	0.9	0.78–0.98	h_1_10	0.17	0.3		
	h00101	0.15	0.8			h_0_01	0.18	0.3		
	Combined rare		0.4							
Block 2: 07,09,10,22,11	h01010	0.27	0.8			h_1_10	0.27	0.9		
	h01000	0.19	0.05	0.9	0.80–1.00	h_1_00	0.20	0.4		
	h00100	0.18	0.9							
	h10000	0.17	0.8			h_0_00	0.40	0.6		
	h10001	0.07	0.07	1.2	0.99–1.37	h_0_01	0.11	0.2		
	h00000	0.05	0.7							
	Combined rare		0.4							
Block 3: 13,14,16,18,19,21	h000010	0.27	0.6			h0____0	0.57	0.9		
	h000001	0.21	0.1	0.9	0.83–1.02	h0____1	0.22	0.5		
	h100000	0.15	0.04	1.1	1.01–1.27	h1____0	0.21	0.5		
	h010000	0.15	0.2							
	h011000	0.12	0.4							
	h100100	0.06	0.1	1.2	0.98–1.35					
	Combined rare		0.8							
***CHEK2***										
10,11,6,7,5,8,12,13,14,9	h0000000000	0.23	0.3			h_0______00	0.54	0.02	0.9	0.88–0.99
	h1000111000	0.20	0.5							
	h0000000011	0.13	0.2			h_0______11	0.13	0.07	1.1	0.99–1.18
	h0111000010	0.07	0.08	0.9	0.74–1.02	h_1______10	0.07	0.1	0.9	0.81–1.02
	h0000111000	0.07	0.8							
	h0011000010	0.06	0.6			h_0______10	0.25	0.06	1.1	0.99–1.15
	h0010000110	0.06	0.2							
	h0000100010	0.04	0.05	1.3	1.00–1.56					
	Combined rare		0.5			h_0______10 delC	0.004	0.003	2.7	1.40–5.05
***TP53***										
03,01,02,06,07,08	h000000	0.42	0.8							
	h000001	0.28	0.9							
	h011000	0.11	0.3							
	h001001	0.05	0.2							
	Combined rare		0.4							

All haplotypes with predicted frequencies of >0.05 in controls are shown individually. Rarer ones are combined. The single nucleotide polymorphism (SNP) IDs and their order are shown in the first column. CI, confidence interval; OR, odds ratio.
