# Supplementary material for: Common variants in the ATM, BRCA1, BRCA2, CHEK2 and TP53 cancer susceptibility genes are unlikely to increase breast cancer risk
Source: Breast Cancer Res. 2007 Apr 11;9(2):R27. doi: 10.1186/bcr1669 (PMC1868915; doi:10.1186/bcr1669)
Supplement: Additional file 1 — A table listing the basic epidemiological details of the breast cancer cases and controls in set 1 and set 2. [file bcr1669-S1.doc]

Supplementary Table 1

Basic epidemiological details of the breast cancer cases and controls in Set 1 and Set 2

|  |  |  | Age (Range) (IQR) | Time to recruit (months) | Morphology | | | Stage | |
| --- | --- | --- | --- | --- | --- | --- | --- | --- | --- |
|  |  |  |  |  | Ductal | Lobular | Other | I/II | III/IV |
| Set1 | P | 1528 | 52  (26 – 69) (47 – 58) | 5.8  (0 – 60) (4.6–98.9) | 1075 (71%) | 246 (16%) | 186 | 1431 (95%) | 72 |
|  | R | 743 | 48  (25 – 54) (43 – 51) | 38  (9.6– 104) (24 – 59) | 540 (73%) | 107 (14%) | 94 | 668 (95%) | 38 |
|  | All | 2271 | 51  (25 – 69) (45 – 55) | 8.8  (0 – 104) (5.0 – 28) | 1615 (72%) | 353 (16%) | 280 | 2099 (95%) | 110 |
|  | Controls | 2280 | 65  (44 – 81) (59 – 71) |  |  |  |  |  |  |
| Set2 | P | 1743 | 55  (23 – 69) (49 – 63) | 15  (2.0 – 82) (7.1 – 25) | 1295 (75%) | 245 (14%) | 187 | 1643 (96%) | 66 |
|  | R | 460 | 48  (23 – 54) (44 – 51) | 41  (4.6 - 141) (8.0 – 92) | 351 (75%) | 64 (14%) | 45 | 433 (96%) | 19 |
|  | All | 2203 | 52  (23 – 69) (47 – 61) | 15  (2.0 – 82) (7.1 – 25) | 1646 (75%) | 309 (14%) | 232 | 2076 (96%) | 85 |
|  | Controls | 2280 | 59  (42 – 81) (52 – 68) |  |  |  |  |  |  |
| Non-recruited cases | | 4215 | 53  (23 – 69) (47 – 69) |  | 3186 (76%) | 545 (13%) | 461 | 3190 (90%) | 354 |
