# Supplementary material for: Common variants in the ATM, BRCA1, BRCA2, CHEK2 and TP53 cancer susceptibility genes are unlikely to increase breast cancer risk
Source: Breast Cancer Res. 2007 Apr 11;9(2):R27. doi: 10.1186/bcr1669 (PMC1868915; doi:10.1186/bcr1669)
Supplement: Additional file 2 — A table showing a statistical power comparison of the two-stage versus the single-stage study design. [file bcr1669-S2.doc]

Supplementary Table 2 Statistical power comparison of the 2 stage Vs single stage study design

|  | Allele frequency | | | | | | | |
| --- | --- | --- | --- | --- | --- | --- | --- | --- |
|  | Staged design | | | | Genotype all samples | | | |
|  | 0.05 | 0.1 | 0.25 | 0.5 | 0.05 | 0.1 | 0.25 | 0.5 |
| Dominant |  |  |  |  |  |  |  |  |
| 1.3 | 16% | 55% | 87% | 59% | 22% | 60% | 89% | 64% |
| 1.5 | 86% | 100% | 100% | 99% | 88% | 100% | 100% | 100% |
| 2.0 | 100% | 100% | 100% | 100% | 100% | 100% | 100% | 100% |
| Recessive |  |  |  |  |  |  |  |  |
| 1.3 | 0% | 0% | 5.0% | 71% | 0% | 0% | 9% | 75% |
| 1.5 | 0% | 0.3% | 53% | 100% | 0% | 1% | 60% | 100% |
| 2.0 | 0.2% | 14% | 100% | 100% | 1% | 21% | 100% | 100% |
